# Integrated transcriptome and small RNA sequencing analyses reveal a drought stress response network in *Sophora tonkinensis*

**DOI:** 10.1186/s12870-021-03334-6

**Published:** 2021-12-02

**Authors:** Ying Liang, Kunhua Wei, Fan Wei, Shuangshuang Qin, Chuanhua Deng, Yang Lin, Mingjie Li, Li Gu, Guili Wei, Jianhua Miao, Zhongyi Zhang

**Affiliations:** 1 College of Agriculture, Fujian Agriculture and Forestry University, No. 15 Shangxiadian Road, Cangshan District, Fuzhou, 350002 People’s Republic of China; 2 Guangxi key Laboratory of Medicinal Resources Protection and Genetic Improvement, Guangxi Botanical Garden of Medicinal Plants, No. 189 Changgang Road, Xingning District, Nanning, 530023 People’s Republic of China; 3 Guangxi Engineering Research Center of TCM Resource Intelligent Creation, Guangxi Botanical Garden of Medicinal Plants, Nanning, 530023 China; 4 Guangxi Forest Inventory and Planning Institute, Nanning, 530011 China; 5 Key Laboratory of Genetics, Breeding and Comprehensive Utilization of Crops, Ministry of Education, Fujian Agriculture and Forestry University, Fuzhou, 350002 China

**Keywords:** *Sophora tonkinensis*, Drought stress, mRNA, miRNA, Next-generation sequencing, Transcription factor

## Abstract

**Background:**

*Sophora tonkinensis* Gagnep is a traditional Chinese medical plant that is mainly cultivated in southern China. Drought stress is one of the major abiotic stresses that negatively impacts *S. tonkinensis* growth. However, the molecular mechanisms governing the responses to drought stress in *S. tonkinensis* at the transcriptional and posttranscriptional levels are not well understood.

**Results:**

To identify genes and miRNAs involved in drought stress responses in *S. tonkinensis*, both mRNA and small RNA sequencing was performed in root samples under control, mild drought, and severe drought conditions. mRNA sequencing revealed 66,476 unigenes, and the differentially expressed unigenes (DEGs) were associated with several key pathways, including phenylpropanoid biosynthesis, sugar metabolism, and quinolizidine alkaloid biosynthesis pathways. A total of 10 and 30 transcription factors (TFs) were identified among the DEGs under mild and severe drought stress, respectively. Moreover, small RNA sequencing revealed a total of 368 miRNAs, including 255 known miRNAs and 113 novel miRNAs. The differentially expressed miRNAs and their target genes were involved in the regulation of plant hormone signal transduction, the spliceosome, and ribosomes. Analysis of the regulatory network involved in the response to drought stress revealed 37 differentially expressed miRNA-mRNA pairs.

**Conclusion:**

This is the first study to simultaneously profile the expression patterns of mRNAs and miRNAs on a genome-wide scale to elucidate the molecular mechanisms of the drought stress responses of *S. tonkinensis*. Our results suggest that *S. tonkinensis* implements diverse mechanisms to modulate its responses to drought stress.

**Supplementary Information:**

The online version contains supplementary material available at 10.1186/s12870-021-03334-6.

## Background

*Sophora tonkinensis* is a traditional Chinese plant included in the “*Pharmacopoeia of the People’s Republic of China* (2020 edition)”, and the roots and rhizomes (known as “Shan-Dou-Gen” in Chinese) of this plant have been used as folk medicine for the treatment of fever, throat inflammation, and tumours [1]. *S. tonkinensis* is the main raw material of greater than 30 types of Chinese patent drugs. *S. tonkinensis* is also used as a decoction piece in many prescription medicines, and has been applied for one thousand years. The species belongs to the family Leguminosae, and its natural habitat is mainly distributed in karst areas of southern China and northern Vietnam [2]. The type of environment that is suitable for *S. tonkinensis* growth is mountainous regions with rocky or limestone substrates [3]. As a result of overexploitation and habitat degeneration, the wild resources of *S. tonkinensis* have decreased rapidly over recent years or even become extinct in many distribution areas. However, the market demand for *S. tonkinensis* is gradually increasing. Thus, cultivated *S. tonkinensis* has become the main source of *S. tonkinensis*-based drugs. The main cultivation areas of *S. tonkinensis* in China are Guangxi, Guizhou, and Yunnan provinces, among which Guangxi has the largest cultivation area of 1334 ha. The annual yield of *S. tonkinensis* in Guangxi is approximately 2442 tons. As the demand for *S. tonkinensis*-based drugs is ever increasing, the price of *S. tonkinensis* roots increased from approximately 23.05 dollars/kg in 2016 [4] to approximately 31.73 dollars/kg in 2020.

More than 150 chemical components have been isolated from *S. tonkinensis*, including flavonoids, alkaloids, polysaccharides, volatile oils, and small amounts of triterpenoids and phenols [1, 5]. Phytochemical studies on *S. tonkinensis* have shown that quinolizidine alkaloids and flavonoids are the principal constituents of the plant. Matrine, one of the quinolizidine alkaloids found in the roots of *S. tonkinensis*, acts as the main medicinal active ingredient and has been proven to have anti-inflammatory, antibacterial, antioxidant, immunomodulatory, and anticancer effects [6]. However, the molecular mechanisms underlying the biosynthesis of quinolizidine alkaloids are not well understood.

The karst areas in southern China where *S. tonkinensis* is mainly distributed have experienced extremely variable conditions related to hydrologic droughts in recent decades [7]. Climate change models predict more frequent incidences of drought and extreme temperature in the near future [8]. *S. tonkinensis* is relatively tolerant to drought stress. Mild drought stress promotes the accumulation of matrine and oxymatrine in the roots of *S. tonkinensis* [9]. However, severe drought stress may cause excess superoxide production in the chloroplast, resulting in photoinhibition and photooxidation damage [10]. Due to the endangered status of wild *S. tonkinensis*, improving plant resilience to drought stress and protecting its medicinal active ingredients has become a major target for breeding programs.

In non-model plants without a reference genome, such as *S. tonkinensis*, mRNA sequencing approaches provide a rapid method for improving drought tolerance through genetic manipulation [11]. Numerous genes that are differentially regulated under drought stress can be rapidly identified through mRNA sequencing. Drought stress in soybean was shown to result in the differential expression of a total of 6609 transcripts, including many genes related to hormones (auxin/ethylene), carbohydrates, cell wall-related secondary metabolism, and transcription factors (TFs) controlling root growth [12, 13]. mRNA sequencing is also a powerful tool for discovering novel genes that participate in secondary metabolite biosynthesis. In *Ginkgo biloba* L., 23 bHLH, 9 MYB, 5 WRKY, and 4 bZIP genes that act as regulators in flavonoid and terpenoid biosynthesis were identified through mRNA sequencing [14]. In *S. tonkinensis*, however, the application of mRNA sequencing to identify drought-related genes has not been reported. Only one recent study utilized mRNA sequencing to study genes involved in flower development in this species [15].

Plant miRNAs can rapidly respond to different developmental and environmental signals and subsequently suppress the expression of their targets via mRNA cleavage or transcriptional inhibition [16–18]. In addition to mRNA sequencing, small RNA sequencing is another powerful high-throughput method for investigating stress regulatory networks. Many studies have shown that miRNAs are involved in regulating a wide range of plant stress resistance processes, including abiotic stress and biotic stress responses [19, 20]. For instance, among the 1643 miRNAs identified through sequencing in Tibetan wild barley, 12 miRNAs were regarded as drought tolerance-associated miRNAs [21]. In sweet potato leaves and roots, many miRNAs were significantly differentially regulated by salinity stress. Some of these miRNAs functioned in a tissue-specific manner in sweet potato under salinity stress [22]. Integrated small RNA sequencing and transcriptome analyses revealed drought stress regulatory networks in durum wheat and tomato [23, 24]. In durum wheat, key miRNA-mRNA modules (particularly, novel pairs of miRNAs and transcription factors) with antagonistic regulatory patterns were identified in response to drought and heat stresses [23]. In tomato, target genes were retrieved from 47 out of the 54 differentially-expressed conserved miRNAs, many of which were related to drought stress tolerance [24].

Although drought stress-related mRNAs and miRNAs have been documented in many plant species, information regarding miRNAs and their targets in *S. tonkinensis* under drought stress conditions is scarce. Here, we report the first systematic investigation of the regulatory networks of *S. tonkinensis* under drought stress based on the high-throughput sequencing of mRNAs and miRNAs. The data presented here will help elucidate the genes and miRNAs involved in drought tolerance and secondary metabolite synthesis in *S. tonkinensis* and provide novel insights into the associated molecular mechanisms, as a resource for breeding to improve drought tolerance in cultivated *S. tonkinensis*. In addition, this study provides useful information for the application of *S. tonkinensis* roots in the pharmaceutical industry.

## Results

### Physiological changes in *S. tonkinensis* under drought stress

**Fig. 1:**
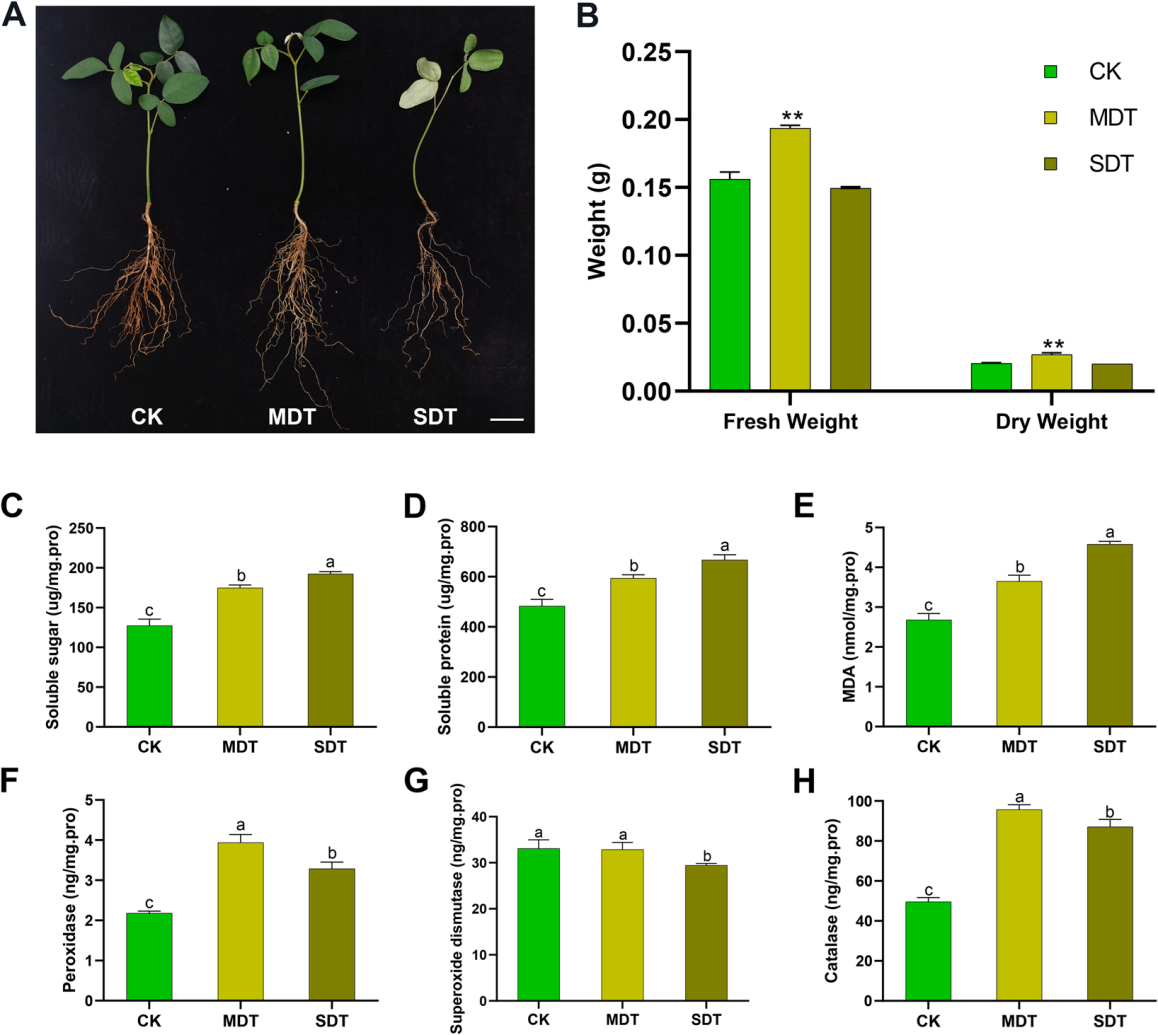
*S. tonkinensis* phenotypic responses to different irrigation treatments. Morphological traits of *S. tonkinensis* under three irrigation treatments: control treatment (CK), mild drought treatment (MDT), and severe drought treatment (SDT) (**A**). Bar = 20 mm. The effects of drought treatment on root fresh weight (g) and dry weight (g) in CK, MDT, and SDT (**B**). Effect of drought treatment on soluble sugar content (**C**), soluble protein content (**D**), MDA content (**E**), peroxidase activity (**F**), superoxide dismutase activity (**G**), and catalase activity (**H**). Each bar represents the mean ± standard error, *n* = 3. ** indicates a significant difference at *p* < 0.01 compared with CK using a two-tailed Student’s t-test. Different letters represent significant differences at *p* < 0.05 (LSD test)

To study the physiological changes in *S. tonkinensis* under drought stress, plant roots were collected under three irrigation treatments: control treatment (CK), mild drought treatment (MDT), and severe drought treatment (SDT) (Fig. 1A). The SDT plants exhibited severe wilting of leaves after 10 days of severe drought stress. The physiological parameters of fresh weight and dry weight were measured in root samples. Significant increases in fresh weight and dry weight were observed in MDT relative to CK (*p* < 0.01). However, no significant difference in fresh weight or dry weight was noted between CK and SDT (Fig. 1B). Under drought stress, the contents of soluble sugar, soluble protein, and malondialdehyde (MDA) were significantly higher than those in CK. The activities of peroxidase and catalase were also significantly increased under drought stress compared with CK. Furthermore, the soluble sugar content, soluble protein content, and MDA content were significantly higher in SDT than in MDT (Fig. 1C-E). In contrast, peroxidase and catalase activities were significantly increased in MDT compared with SDT (Fig. 1F-H). To elucidate the genes and miRNAs involved in the drought response mechanisms of *S. tonkinensis*, the collected root samples were subjected to next-generation sequencing to identify DEGs and differentially expressed miRNAs (DEMs).

### De novo analysis of the *S. tonkinensis* root transcriptome

A total of 9 RNA libraries from the three irradiation regimens were prepared and analysed. After removing the reads with linkers and low-quality reads, 20.45-22.59 million clean reads (6.12-6.76 Gb in total) were obtained. The GC content ranged from 43.98-44.26%, and Q30 ranged from 92.59-93.94% (Table 1). The de novo assembly of the clean reads produced a total of 66,476 unigenes (75.0 Mb in size) with a mean length of 1128 bp and an N50 length of 1914 bp. A total of 331,534 transcripts (603.7 Mb in size) were obtained, with a mean length of 1821 bp and an N50 length of 2508 bp. Furthermore, a total of 11,150 (16.77%) unigenes and 122,940 (37.08%) transcripts were longer than 2000 bp (Table 2).

**Table 1:** Summary of transcriptome sequencing results after filtering

Sample^a^	Sample ID	Total Clean Reads^b^	Total Clean Bases (bp)	GC (%)^c^	Q30 (%)^d^
Control Treatment	CK1	21,383,749	6,393,162,008	44.04%	92.94%
	CK2	22,307,167	6,679,646,774	44.22%	93.09%
	CK3	22,434,089	6,715,391,360	44.26%	93.94%
Mild Drought Treatment	MDT1	21,674,654	6,490,540,766	44.20%	93.51%
	MDT2	21,314,973	6,375,756,816	43.98%	92.90%
	MDT3	21,977,880	6,581,391,768	44.17%	93.33%
Severe Drought Treatment	SDT1	20,449,638	6,120,514,738	44.25%	93.15%
	SDT2	22,588,355	6,762,437,920	44.22%	93.64%
	SDT3	22,504,624	6,736,056,616	44.11%	92.59%

^a^ For each treatment, three independent biological replicates were collected and sequenced

^b^ Total clean reads were obtained after removing the reads containing linkers and low-quality reads

^c^ GC content of the bases

^d^ Q30: the percentage of bases with a Phred quality score greater than 30

**Table 2:** Overview of the assembled transcriptome

Length Range^a^	Transcript	Unigene
300-500	44,401 (13.39%)	25,341 (38.12%)
500-1000	64,453 (19.44%)	18,784 (28.26%)
1000-2000	99,740 (30.08%)	11,201 (16.85%)
2000+	122,940 (37.08%)	11,150 (16.77%)
Total Number	331,534	66,476
Total Length	603,712,687	74,997,502
N50 Length	2508	1914
Mean Length	1821	1128

^a^ Length distribution of the assembled transcripts and unigenes

**Fig. 2:**
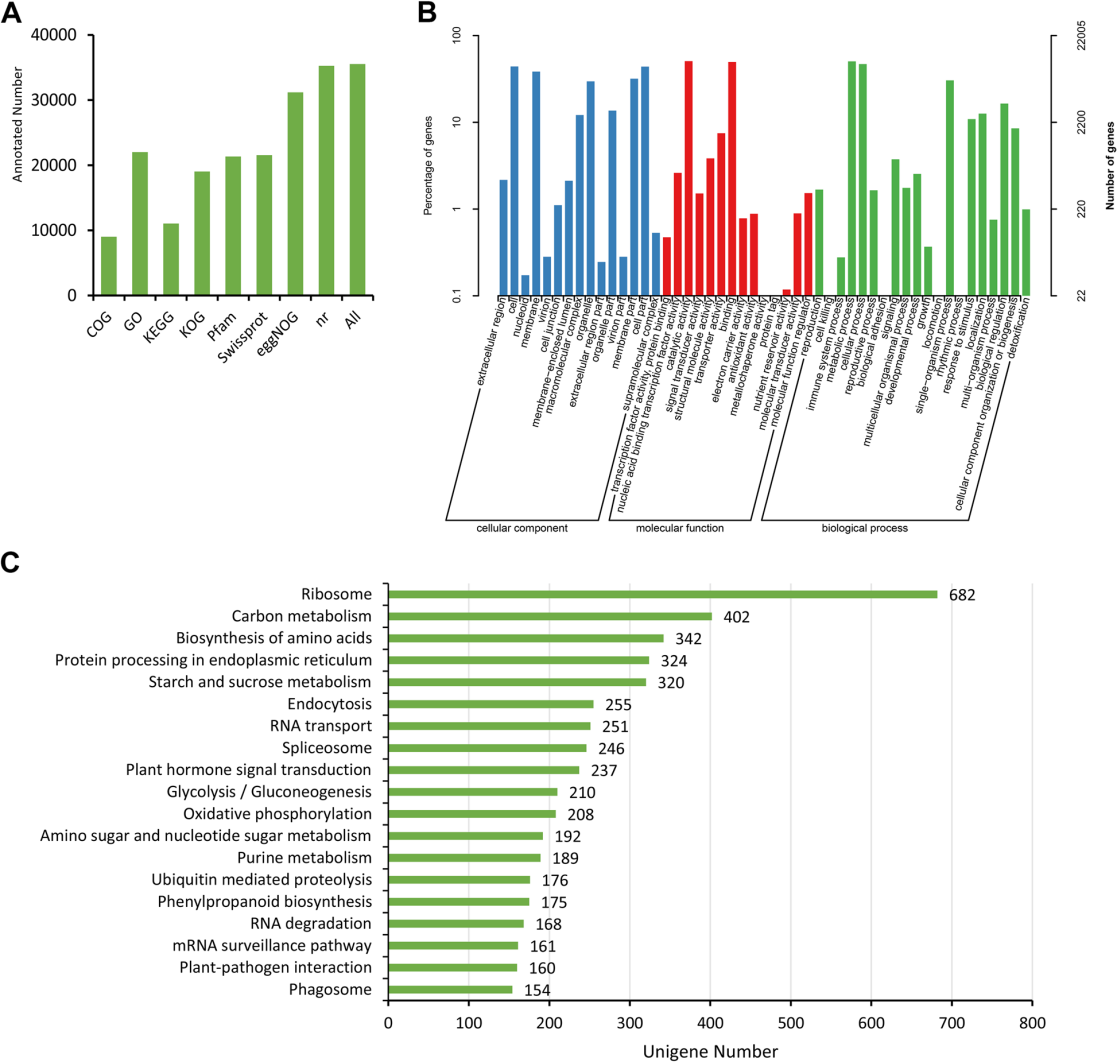
Unigene annotation results. Annotation information obtained from eight different databases (**A**). Annotation information based on the Gene Ontology database (**B**). The most common KEGG pathways involved more than 150 unigenes. The top 19 out of 129 KEGG pathways are presented in this Fig. (**C**)

The unigenes obtained from the assembly were annotated by BLAST alignment with eight public databases (COG, GO, KEGG, KOG, Pfam, Swiss-Prot, eggNOG, and Nr). In total, 35,537 unigenes (53.46% of all unigenes) were annotated according to showing matches in at least one of the databases. A total of 9014 (13.56%), 22,005 (33.10%), 11,055 (16.63%), 19,031 (28.63%), 21,332 (32.09%), 21,552 (32.42%), 31,185 (46.91%), and 35,267 (53.05%) unigenes were annotated in the COG, GO, KEGG, KOG, Pfam, Swiss-Prot, eggNOG and Nr databases, respectively (Fig. 2A).

### GO classification and KEGG pathway analysis

GO terms associated with each unigene were identified based on sequence homology. A total of 22,005 unigenes were classified into 49 GO terms, which were assigned to the three main categories of biological processes (BPs), molecular functions (MFs), and cellular components (CCs). In the BP category, the unigenes were further divided into 20 terms, among which “metabolic process” (11,095), “cellular process” (10,311), and “single-organism process” (6700) were the main GO terms. In the MF category, the unigenes were further divided into 14 terms. The greatest numbers of unigenes (11,153 and 10,919) were annotated to “catalytic activity” and “binding” terms, respectively. In the CC category, the unigenes were further divided into 15 terms, with “cell” (9685), “cell part” (9649), and “membrane” (8448) representing the main GO terms (Fig. 2B). Additionally, a total of 27 unigenes were involved in the BP of the response to water deprivation (GO: 0009414).

The KEGG database was used to identify 129 important pathways involved in various developmental processes in plants (Fig. 2C). Among these pathways, the “ribosome” (682), “carbon metabolism” (402), and “biosynthesis of amino acids” (342) pathways included markedly higher numbers of unigenes than the other pathways. Some of the key pathways involving the accumulation of medicinal compounds in *S. tonkinensis*, such as the “flavonoid biosynthesis” (40), “tropane, piperidine and pyridine alkaloid biosynthesis” (26), and “isoquinoline alkaloid biosynthesis” (23) pathways, also included a significant number of expressed unigenes.

### Analysis of differential gene expression

**Fig. 3:**
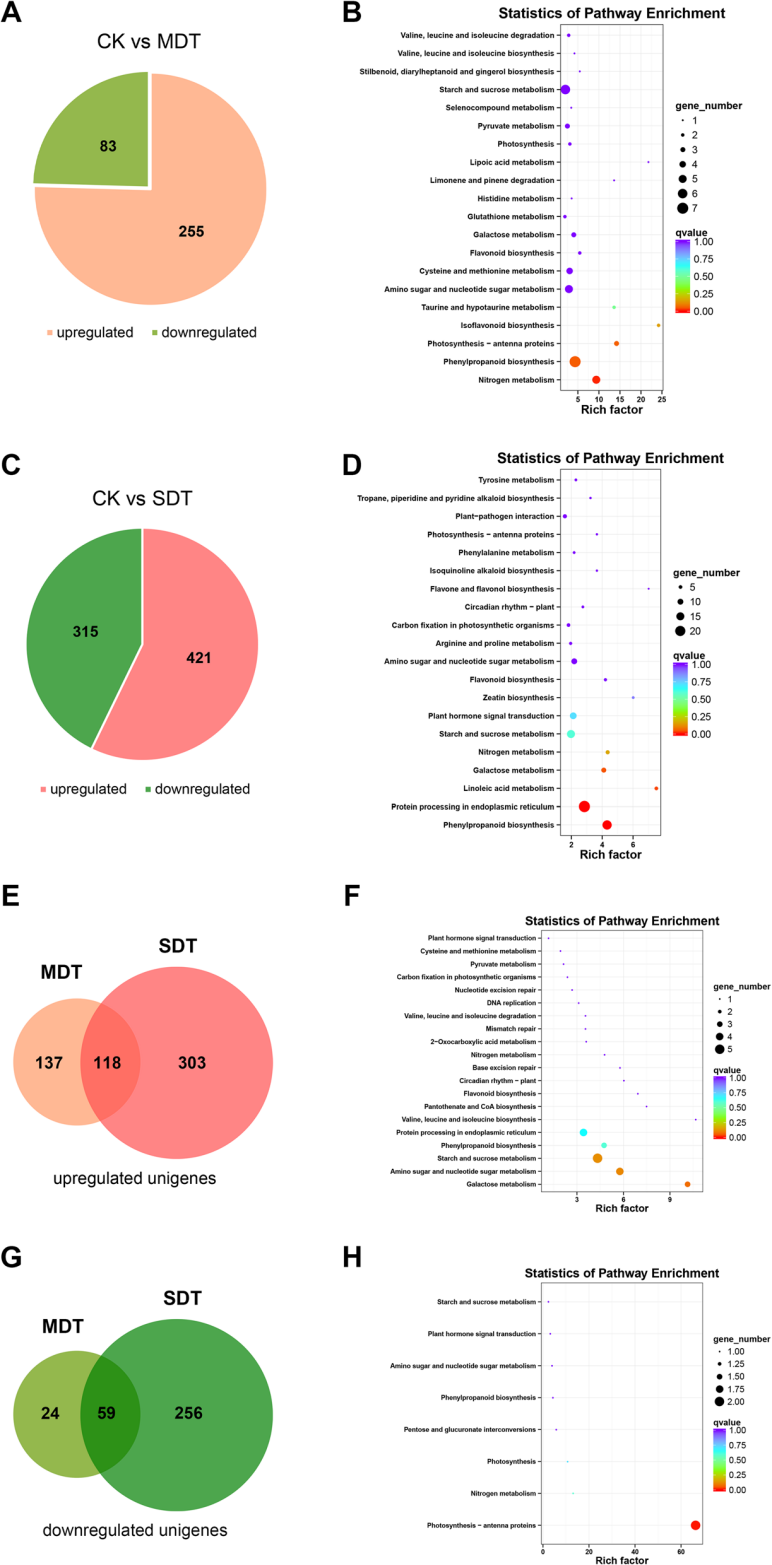
Analysis of differential gene expression and KEGG enrichment. Differentially regulated genes under mild drought treatment (MDT) (**A**) and severe drought treatment (SDT) (**C**) compared with the control condition (CK). KEGG enrichment analysis of the DEGs under mild drought treatment (**B**) and severe drought treatment (**D**). Venn diagrams showing the numbers of specific and common upregulated (**E**) and downregulated (**G**) unigenes. KEGG enrichment analysis of the co-upregulated (**F**) and co-downregulated (**H**) unigenes

DEGs under the different irrigation treatments were examined. Compared with CK, MDT showed 338 DEGs, among which 255 were upregulated and 83 were downregulated (FDR < 0.01 and |log2 fold-change| ≥ 1) (Fig. 3A, Table S2). Among the 338 DEGs sequenced from the MDT samples, 56 DEGs were annotated to 53 metabolic pathways in the KEGG database (Fig. 3B). Among these pathways, “nitrogen metabolism” was significantly enriched (corrected *p*-value: 0.009124), with 4 upregulated unigenes and 1 downregulated unigene in the pathway. The “phenylpropanoid biosynthesis” pathway presented the highest number of differentially expressed unigenes, with 6 upregulated unigenes and 1 downregulated unigene.

Significantly more DEGs (736) were identified in SDT than in CK, among which 421 were upregulated and 315 were downregulated (Fig. 3C, Table S2). Among the 736 DEGs sequenced from the SDT samples, 145 were annotated to 69 metabolic pathways in the KEGG database (Fig. 3D). Among these pathways, 4 were significantly enriched (corrected *p*-values less than 0.05), including “phenylpropanoid biosynthesis”, “protein processing in endoplasmic reticulum”, “linoleic acid metabolism” and “galactose metabolism”. The phenylpropanoid biosynthesis pathway was the most significantly enriched (corrected *p*-value: 0.00000880), with 5 upregulated unigenes and 13 downregulated unigenes in the pathway. Interestingly, 3 upregulated unigenes and 1 downregulated unigene from the phenylpropanoid biosynthesis pathway identified in MDT were consistently upregulated or downregulated in SDT. The remaining 3 upregulated unigenes identified in MDT were not significantly differentially regulated in SDT. Flavonoids are a class of secondary metabolites formed by phenylpropanoid biosynthesis pathways in higher plants. Flavonoids are abundant in legumes. The contents of two main flavonoids in *S. tonkinensis*, genistein and maackiain, were measured using high-performance liquid chromatography (HPLC). The genistein and maackiain contents in the MDT were significantly higher than those in the CK (Fig. S2).

The “protein processing in endoplasmic reticulum” pathway was also significantly enriched (corrected *p*-value: 0.000454), with 19 upregulated unigenes and 3 downregulated unigenes in the pathway. In addition, many upstream pathways related to protein accumulation, such as the “nitrogen metabolism” pathway, “arginine and proline metabolism” pathway, “valine, leucine and isoleucine biosynthesis” pathway, and “biosynthesis of amino acids” pathway, were all present in the list of enriched pathways of DEGs.

Among the DEGs, 118 unigenes were upregulated under both MDT and SDT (Fig. 3E), whereas 59 unigenes were downregulated under both MDT and SDT (Fig. 3G). The 118 co-upregulated unigenes were mainly enriched in three major KEGG pathways: “galactose metabolism”, “amino sugar and nucleotide sugar metabolism”, and “starch and sucrose metabolism” (Fig. 3F). A total of 7 co-upregulated unigenes were included in the three pathways, as these three pathways share many common genes. The upregulated unigenes involved in these KEGG pathways encode 3 beta-fructofuranosidases, 2 chitinases, and 2 glucose-1-phosphate adenylyltransferases. In addition to the unigenes mentioned above, some additional unigenes were co-upregulated, including c88287.graph_c0 in the flavonoid biosynthesis pathway. c88287.graph_c0 encodes a chalcone synthase (CHS) protein that functions in the first step of flavonoid biosynthesis to catalyse the transformation of p-coumaroyl-CoA into chalcone. Among the 118 co-upregulated unigenes, 59 were annotated to 26 GO terms. The most common GO terms in the BP, CC, and MF categories were “metabolic process” (27), “cell” (26), and “catalytic activity” (29), respectively (Fig. S1A). The 59 co-downregulated unigenes were significantly enriched in the photosynthesis-antenna protein pathway, which included only 2 unigenes (Fig. 3H). Among the 59 co-downregulated unigenes, 35 were annotated to 18 GO terms. The most common GO terms in the BP, CC, and MF categories were “metabolic process” (16), “cell” (14), and “catalytic activity” (16), respectively (Fig. S1B).

### Differential expression of TFs

A total of 1096 TFs from 65 TF families, such as AP2/ERF (101), C2H2 (96), MYB-related (75), bHLH (70), and bZIP (56), were identified among the unigenes. Among the 338 DEGs from MDT compared to CK, 10 unigenes encoded TFs, including 5 upregulated and 5 downregulated TFs. These TFs were classified into 8 different families, including the AP2/ERF (2 unigenes) and bZIP (2 unigenes) families and 6 additional families that each contained only 1 unigene (WRKY, Trihelix, RWP-RK, MADS-M-type, FAR1, and C2H2). Among the 736 DEGs identified under SDT compared to CK, 30 unigenes encoded TFs, including 11 upregulated and 19 downregulated TFs. These TFs were classified into 11 different families, including the bHLH (6 unigenes), AP2/ERF (4), WRKY (4), MYB-related (3), C2H2 (3), NAC (3), bZIP (3), HB-HD-ZIP (1), MADS-M-type (1), FAR1 (1), and GARP-G2-like (1) families.

**Fig. 4:**
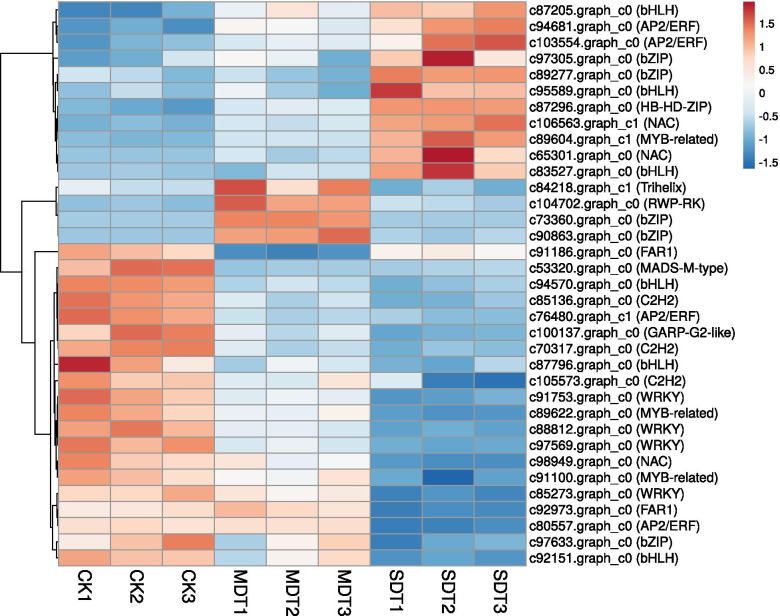
Hierarchical clustering of 35 significantly differentially expressed TFs under mild or severe drought treatment. CK: control; MDT: mild drought treatment; SDT: severe drought treatment

In total, 35 TFs were significantly differentially expressed in either MDT or SDT (Fig. 4). Interestingly, the 4 WRKY TFs and 3 C2H2 TFs were downregulated in both MDT and SDT. We obtained 49 WRKY TF unigenes in *S. tonkinensis,* one of which, StWRKY72 (c97569.graph_c0), was downregulated by 2- and 4-fold in MDT and SDT, respectively. The other three WRKY TF unigenes, StWRKY9 (c91753.graph_c0), StWKRY27 (c88812.graph_c0), and StWRKY29 (c85273.graph_c0), were slightly downregulated in MDT and significantly downregulated in SDT. C2H2 zinc finger proteins play important roles in plant responses to a wide spectrum of stress conditions, such as cold, salinity, and drought stress [25]. A total of 96 C2H2 zinc finger unigenes were obtained in *S. tonkinensis*, ranking second among the TF families. StZAT11 (c70317.graph_c0) encodes a C2H2 zinc finger protein and was downregulated by 2- and 3-fold in MDT and SDT, respectively.

AP2/ERF TFs regulate responses to numerous abiotic stresses, such as drought, cold, heat, salt, and freezing [26]. There were 101 unigenes encoding AP2/ERF TFs in *S. tonkinensis*, among which two AP2/ERF TFs were differentially expressed in both MDT and SDT. StDREB2 (c94681.graph_c0) was upregulated by 2- and 3.8-fold in MDT and SDT, respectively. In contrast, the other AP2/ERF TF, StERF1 (c76480.graph_c1), was downregulated by 3.2- and 2.3-fold in MDT and SDT, respectively.

A total of 25 MADS-box-type TFs were identified, only one of which was significantly differentially expressed under drought stress. This protein, StAGL80 (c53320.graph_c0), was downregulated by greater than 2.2-fold in both MDT and SDT.

### Genes involved in quinolizidine alkaloid biosynthesis

**Fig. 5:**
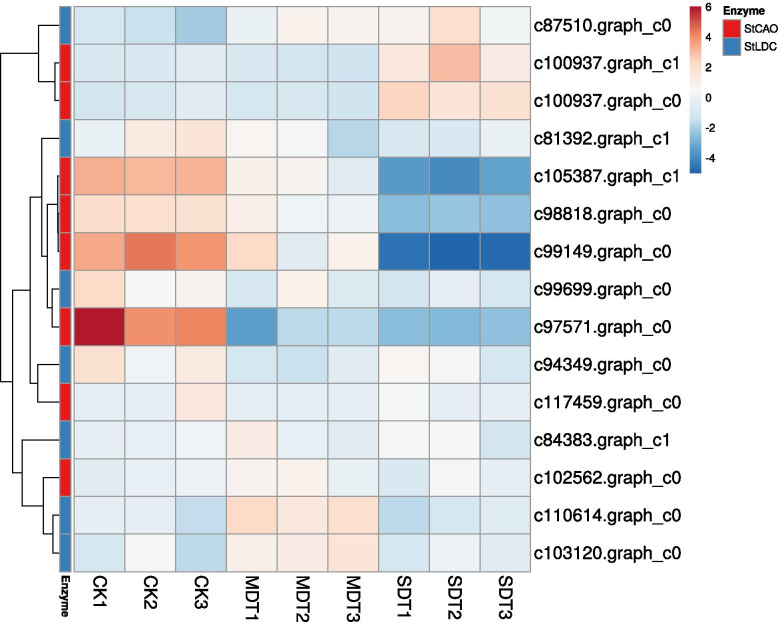
Hierarchical clustering of *StLDCs* and *StCAOs* in the control, mild and severe drought treatments. CK: control; MDT: mild drought treatment; SDT: severe drought treatment

The unigenes assembled from the transcriptome were examined to identify quinolizidine alkaloid (QA) biosynthesis candidate unigenes. Lysine decarboxlylase (LDC) catalyses the first step in the QA pathway in which L-lysine is converted into cadaverine. A total of 7 unigenes encoding LDC enzymes were identified. All of these unigenes were expressed in CK, MDT, and SDT. Moreover, the expression levels of 6 unigenes were not significantly altered in the drought treatment groups relative to the CK group. One LDC unigene (c87510.graph_c0) was upregulated by 1.7- and 2.2-fold in MDT and SDT, respectively. Copper amine oxidase (CAO) catalyses the second step in the QA pathway to convert cadaverine into 5-aminopentanal. The analysis identified 8 CAO unigenes in the transcriptome, among which the expression levels of c99149.graph_c0 were highest, with fragments per kilobase of exon per million mapped fragments (FPKM) values of 165, 153, and 131 in CK, MDT, and SDT, respectively. Two CAOs, c100937.graph_c1 and c105387.graph_c1, were up- and downregulated in SDT by 2.0- and 2.5-fold, respectively (Fig. 5). The contents of two main quinolizidine alkaloids in *S. tonkinensis*, matrine and oxymatrine, were measured using HPLC. Drought stress significantly promoted the accumulation of matrine and oxymatrine, especially in MDT. The matrine content in MDT increased by greater than 1.5-fold compared to CK (Fig. S3).

### De novo analysis of *S. tonkinensis* root miRNAs

**Fig. 6:**
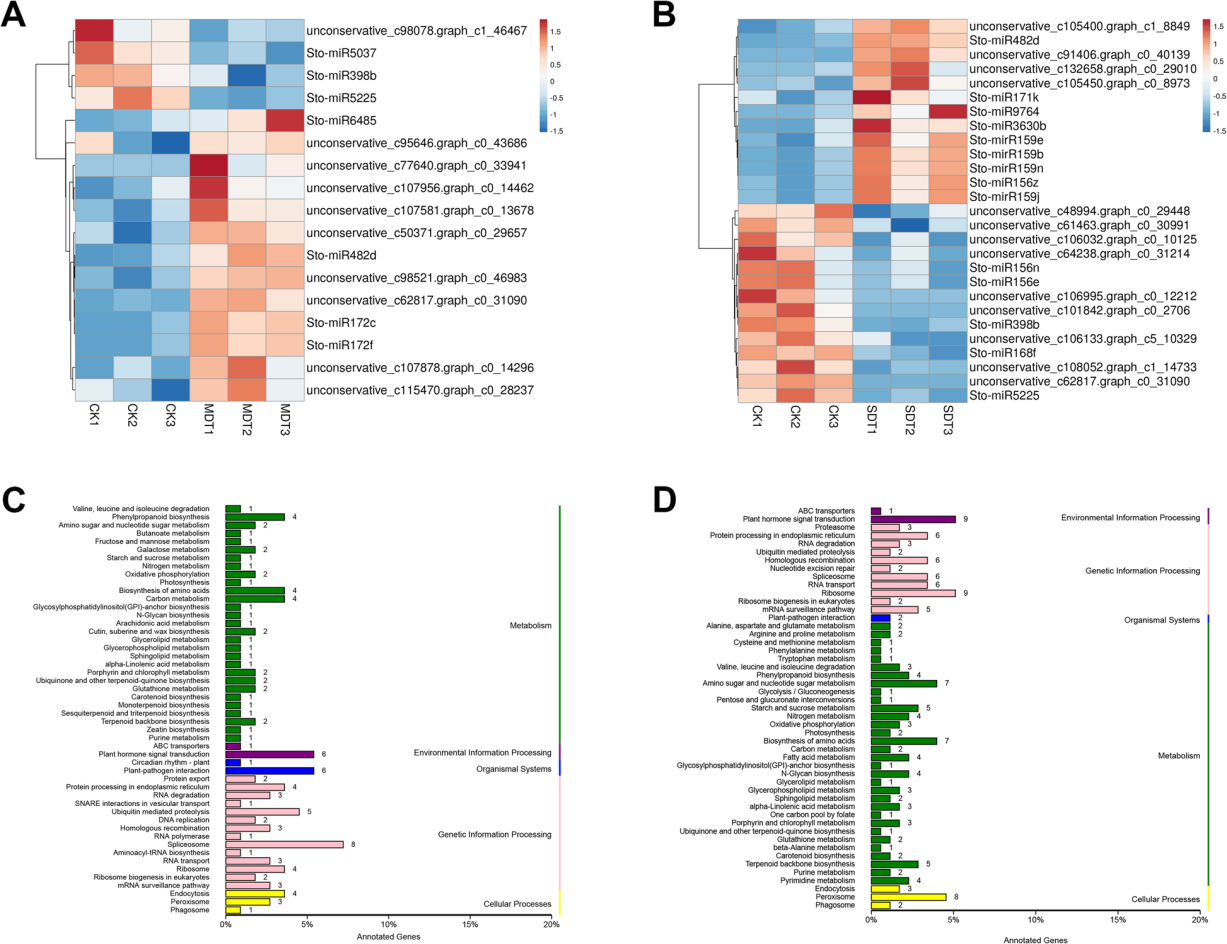
Differentially expressed miRNAs and KEGG annotation of target unigenes. Hierarchical clustering of DEMs in MDT (**A**) and SDT (**B**). KEGG annotation of target unigenes of DEMs in MDT (**C**) and SDT (**D**)

The 9 samples used for transcriptome analysis were also used for miRNA sequencing. After filtration, 6.03-13.62 million clean reads were obtained in these libraries (Table 3). These clean data were mapped onto the assembled unigenes of *S. tonkinensis,* and a total of 368 miRNAs were identified, including 255 known miRNAs and 113 novel miRNAs (Table S3). Moreover, a total of 10,840 target genes were identified for the 368 miRNAs. Comparisons with CK revealed 17 and 27 DEMs in MDT and SDT, respectively (*p*-value < 0.05 and |log2 fold-change| ≥ 0.585). Among these genes, 13 significantly upregulated and 4 downregulated miRNAs were found in MDT (Fig. 6A). In SDT, 13 significantly upregulated and 14 downregulated miRNAs were found (Fig. 6B).

**Table 3:** Summary of miRNA sequencing results after filtering

Sample ID^a^	Total Clean Reads	Mapped Reads^b^	Mapping Ratio (%)
CK1	10,988,901	1,435,715	13.07%
CK2	9,293,459	1,169,253	12.58%
CK3	13,624,014	1,721,199	12.63%
MDT1	7,876,298	1,045,212	13.27%
MDT2	10,221,869	1,174,378	11.49%
MDT3	11,118,692	1,392,929	12.53%
SDT1	6,028,884	784,710	13.02%
SDT2	7,023,727	827,818	11.79%
SDT3	6,307,372	804,659	12.76%

^a^ CK: control; MDT: mild drought treatment; SDT: severe drought treatment

^b^ The clean reads were mapped onto the assembled unigenes

To characterize the regulatory roles of miRNAs in drought stress, DEM target prediction and KEGG annotation analyses were performed. A total of 896 and 1389 target unigenes for the DEMs were identified in MDT and SDT, respectively. For the target unigenes found in MDT, the most common KEGG pathways were “spliceosome”, “plant-pathogen interaction”, and “plant hormone signal transduction” (Fig. 6C). For the target unigenes found in SDT, the most common KEGG pathways were “plant hormone signal transduction”, “ribosome”, and “peroxisome” (Fig. 6D).

To further identify the key miRNAs controlling drought tolerance in *S. tonkinensis*, Venn diagram analysis was performed, and a total of 2 miRNAs that were co-downregulated (Sto-miR398b and Sto-miR5225) and 1 miRNA that was co-upregulated (Sto-miR482d) in both MDT and SDT were identified. For example, the miRNA Sto-miR398b was downregulated by 1.86- and 1.74-fold in MDT and SDT, respectively. Thirteen unigenes were identified as potential targets of Sto-miR398b. In contrast, Sto-miR482d was upregulated by 2.19- and 5.02-fold in MDT and SDT, respectively. A total of 26 unigenes were potential targets of Sto-miR482d.

### Integrated analysis of mRNAs and miRNAs in response to drought stress

**Fig. 7:**
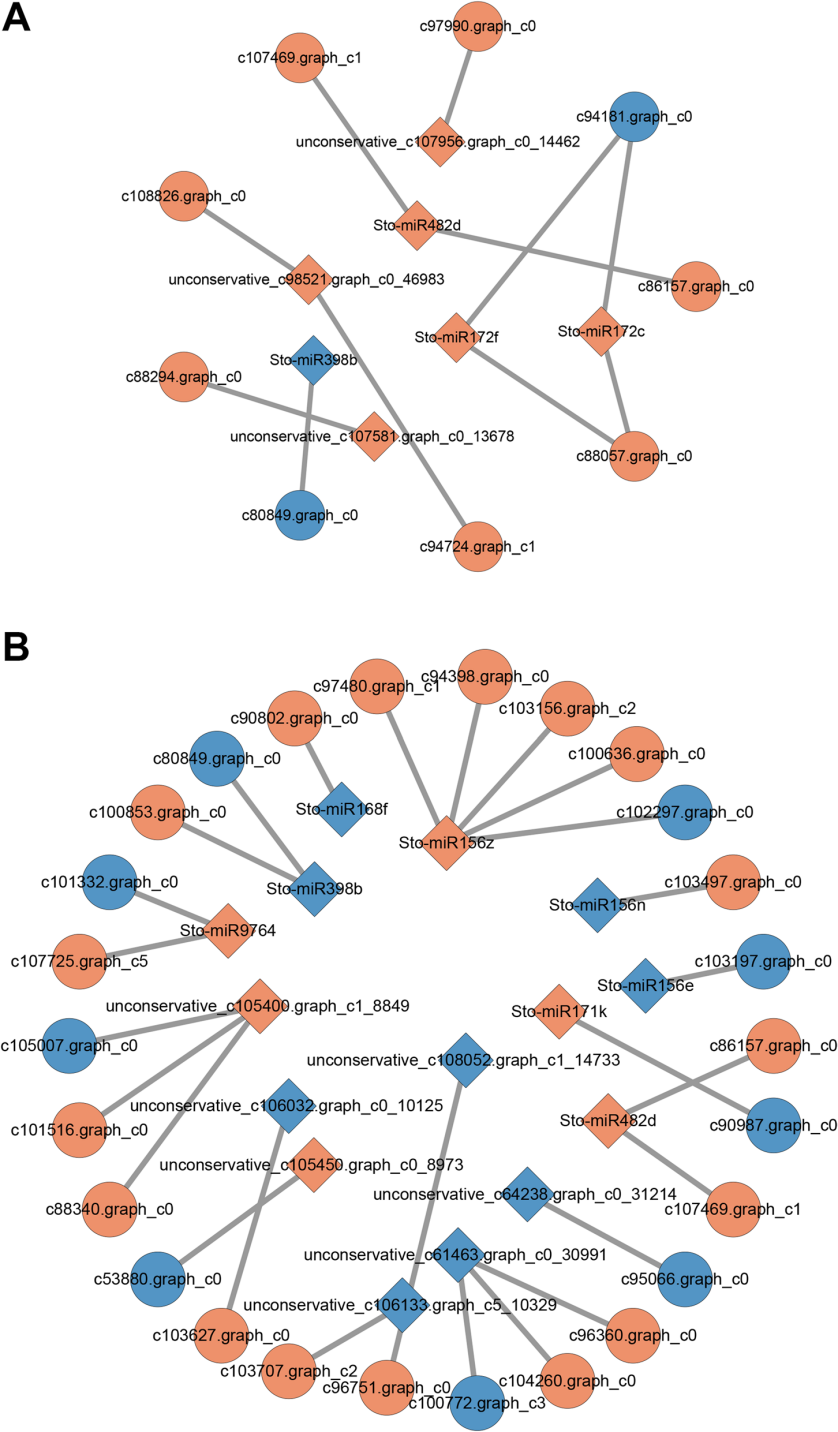
Regulatory network between miRNAs and target mRNAs associated with drought stress in MDT (**A**) and SDT (**B**). Red circles represent upregulated mRNAs, and blue circles represent downregulated mRNAs. Red diamonds represent upregulated miRNAs, and blue diamonds represent downregulated miRNAs

To establish the regulatory network of miRNA-mRNAs involved in the response to drought stress, the potential targets of DEMs were analysed, and common genes with DEGs in the transcriptome were found. In MDT, a total of 11 miRNA-mRNA pairs were found, involving 7 DEMs and 9 DEGs (Fig. 7A). Among these pairs, 8 involved miRNAs and mRNAs that were both upregulated; 1 pair involved a miRNA and mRNA that were both downregulated; and 2 pairs showed antagonistic regulatory patterns involving upregulated miRNAs and downregulated mRNAs. In SDT, a total of 26 miRNA-mRNA pairs were found, involving 15 DEMs and 26 DEGs (Fig. 7B). Among these pairs, 9 involved upregulated miRNAs and mRNAs; 4 pairs involved downregulated miRNAs and mRNAs; 5 pairs involved upregulated miRNAs and downregulated mRNAs; and 8 pairs involved downregulated miRNAs and upregulated mRNAs. Interestingly, miRNA-mRNA pairs in which the miRNA and target mRNA were both upregulated constituted the most common regulatory pattern. For example, miR156z was upregulated in SDT and had 5 target mRNAs, among which 4 were also upregulated. KEGG pathway annotation was found in only 1 and 6 unigenes in the MDT and SDT miRNA-mRNA pairs, respectively. The affected pathways in the miRNA-mRNA regulatory network included the “phenylpropanoid biosynthesis” (c101332.graph_c0), “carotenoid biosynthesis” (c104260.graph_c0), “photosynthesis” (c80849.graph_c0), “fatty acid metabolism” (c90802.graph_c0), “plant hormone signal transduction” (c96751.graph_c0), “glycolysis/gluconeogenesis” and “biosynthesis of amino acids” (c107725.graph_c5) pathways, many of which have been described previously.

### qRT-PCR validation of unigenes and miRNAs

**Fig. 8:**
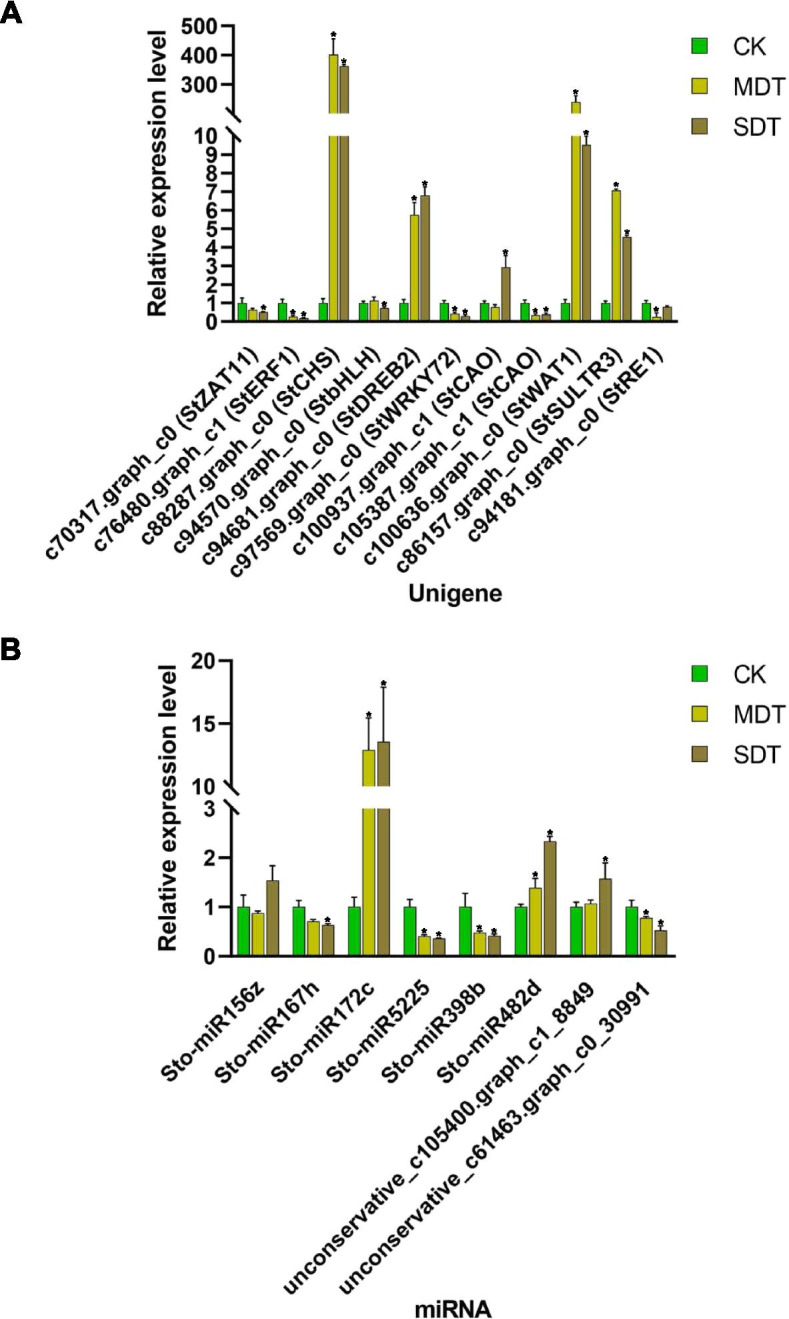
Quantitative real-time PCR (qRT-PCR) validation of selected unigenes and miRNAs. **A** qRT-PCR was performed to determine the expression levels of 11 unigenes relative to actin. **B** The expression of 6 known miRNAs and 2 novel miRNAs was verified by stem-loop qRT-PCR with 18S RNA as an internal reference gene. Error bars indicate ±SD (*n* = 3, from three technical replicates). * indicates a significant difference at *p* < 0.05 compared with CK using a two-tailed Student’s t-test

To verify the reliability of the transcriptome data, the transcript levels of 11 randomly selected unigenes were evaluated by qRT-PCR with three biological replicates. All of these unigenes displayed expression trends that were similar to those obtained in the RNA sequencing analysis, corroborating the RNA sequencing data. The high consistency of the RNA sequencing and qRT-PCR results suggested that the RNA sequencing data were reliable for evaluating the regulation of gene expression in the drought treatment of *S. tonkinensis* (Fig. 8A). To verify the reliability of the small RNA sequencing results, stem-loop qRT-PCR was used to determine the miRNA expression levels of 8 miRNAs with three biological replicates. The stem-loop qRT-PCR results confirmed the expression of seven of the eight miRNAs (with the exception of Sto-miR172c), indicating the reliability of our small RNA sequencing data (Fig. 8B). Among these unigenes and miRNAs, three miRNA-mRNA pairs were selected for verification of regulatory network between miRNAs and target mRNAs (i.e., Sto-miR156z-c100636.graph_c0, Sto-miR482d-c86157.graph_c0, and Sto-miR172c-c94181.graph_c0).

## Discussion

Drought is a major environmental perturbation that affects plant growth and development worldwide. Drought stress is common in karst areas in southern China, where *S. tonkinensis* is mainly distributed [7]. Under drought stress, plants mobilize many defence mechanisms to cope with environmental challenges, which involve molecular, biochemical and physiological changes [27]. In this study, the contents of soluble sugar, soluble protein, and MDA as well as the activities of peroxidase and catalase in *S. tonkinensis* roots all increased significantly under drought stress, thus improving drought tolerance through the regulation of antioxidant ability. In this study, we employed next-generation mRNA and miRNA sequencing to explore the molecular changes in *S. tonkinensis* experiencing mild and severe drought stress. This is the first transcriptome study of *S. tonkinensis* under drought stress performed using next-generation sequencing technology.

### DEGs under drought stress

Through transcriptome analysis, we identified 338 and 736 unigenes that were significantly differentially expressed under drought stress in MDT and SDT, respectively. The results showed that the number of DEGs was significantly higher in SDT than in MDT, suggesting that severe drought induced a stronger response than mild drought. This pattern has also been observed in soybean under different drought stress conditions [13].

Functional analysis showed that DEGs were significantly enriched in the phenylpropanoid biosynthesis pathway. Phenylpropanoids exert a significant effect on plant responses to drought stress [28–30]. Key genes in the phenylpropanoid biosynthesis pathway have previously been shown to be modulated in drought-treated basil (*Ocimum basilicum L.)*, a traditional medicinal plant [28]. We identified 3 upregulated unigenes and 1 downregulated unigene in the phenylpropanoid biosynthesis pathway under both mild and severe drought stress (c103066.graph_c1, c105632.graph_c1, c83751.graph_c0, c90823.graph_c0); all of these unigenes encode caffeic acid 3-O-methyltransferase (COMT). COMT is considered a multifunctional enzyme with high substrate promiscuity, as it methylates caffeoyl and 5-hydroxy coniferyl alcohols, aldehydes and free acids [31]. The expression of the grape COMT gene was activated under drought stress [32]. The COMT genes of *S. tonkinensis* may also participate in tolerance to drought stress. Genistein and maackiain are two main downstream flavonoids of the phenylpropanoid biosynthesis pathway in *S. tonkinensis* [33]. Consistent with the upregulation of genes in the phenylpropanoid biosynthesis pathway, drought stress significantly promoted the accumulation of genistein and maackiain, especially in MDT.

Sugar metabolism plays an essential role in enhancing osmotic regulation, which has been shown to be involved in resistance to drought stress in many plants, such as moso bamboo [34], grape berry [35], and cassava [36]. Drought stress increases the contents of soluble sugar, sucrose, and starch in the roots of soybean seedlings by increasing the activities of sucrose and starch metabolism enzymes [37]. In this study, we identified 118 DEGs that were co-upregulated under MDT and SDT. The three major KEGG pathways enriched in co-upregulated unigenes were all related to sugar metabolism. Moreover, the content of soluble sugar was significantly increased under drought treatment, consistent with the upregulation of sugar metabolism genes. Therefore, sugar metabolism may play an important role in mediating tolerance to drought in *S. tonkinensis*.

Plants have various defence systems in place to repair damage caused by various forms of stress. Osmoregulatory substances and antioxidant enzymes are important physiological compounds for plants to adapt to drought stress [38]. A previous study showed that *Bupleurum chinense* can adapt to drought stress mainly by increasing concentrations of osmoregulatory substances (soluble protein) and increasing the activity of protective enzymes (superoxide dismutase and catalase) [39]. Regarding the increase in soluble protein content in *S. tonkinensis*, we noticed that “the protein processing in endoplasmic reticulum” pathway was significantly enriched under SDT, with 19 upregulated unigenes and 3 downregulated unigenes in the pathway. Many upstream pathways related to protein accumulation were also significantly enriched under drought stress. The activation of these pathways may promote the accumulation of soluble protein under drought stress. Consistent with the increase in peroxidase and catalase activities, a total of 4 peroxidase unigenes and 1 catalase unigene were upregulated, and no downregulated peroxidase or catalase unigene was found in MDT. This finding suggests that many of the gene expression changes after drought treatment are related to physiological responses in *S. tonkinensis* roots.

### TFs involved in the drought response

TFs are involved in the regulation of signal transduction and stress-responsive gene expression [40]. TFs are considered early activated genes that are targeted by phosphatases and protein kinases [41]. Most of the TFs that play important roles in abiotic stress tolerance in plants fall into major TF families (AP2/ERF, NAC, MYB/MYC, WRKY, bZIP, bHLH, and ZFP) [42]. A previous study in *Sophora alopecuroides* functionally annotated a total of 11,936 TF genes from 82 TF families under salt, alkali, and drought stress [43]. In this study, we identified 1096 TFs in *S. tonkinensis,* among which 35 were differentially expressed under drought stress. The majority of drought-related TFs identified in this study were classified into families such as AP2/ERF, WRKY, C2H2, MYB-related, NAC, bZIP, and MADS.

The AP2/ERF family was the largest TF family identified in *S. tonkinensis*, with 2 upregulated and 2 downregulated AP2/ERF TFs under drought stress. AP2/ERF expression is tightly regulated to enable proper stress responses. Gene expression profiling studies have shown that most AP2/ERFs are expressed at low levels under normal conditions, whereas their expression can be induced or repressed by abiotic stresses such as drought [26]. For example, rice OsERF71 positively regulates drought tolerance by altering root architecture through the ABA signalling pathway [44]. In contrast, Arabidopsis RAP2.1 negatively regulates drought tolerance, given that RAP2.1 overexpression results in sensitivity to drought [45]. In the present study, we showed that StDREB2 was upregulated under drought stress. DREB2s have been reported to be induced upon drought treatment and to positively regulate DRE-containing drought-responsive genes such as LEAs [46]. In contrast, StERF1 was downregulated by drought stress. Accordingly, rice OsDERF1 is regulated by drought. Specifically, overexpressing OsDERF1 leads to reduced tolerance to drought stress in rice at the seedling stage, whereas knockdown of OsDERF1 expression confers enhanced tolerance in the seedling and tillering stages [47]. The regulation of the AP2/ERF TFs StDREB2 and StERF1 under drought stress was consistent with findings in other plant species, such as Arabidopsis and rice.

WRKYs are a class of DNA-binding proteins that recognize TTGAC(C/T) W-box elements. WRKY TFs respond to stress by regulating plant secondary metabolites such as alkaloids and terpenes [43]. DEG analysis identified 4 differentially expressed WRKY TFs, which were all downregulated under drought stress. However, WRKY TFs are generally considered to be upregulated under drought stress in many plant species, such as Arabidopsis, rice, sunflower, and soybean [48]. In soybean plants, the expression of four members of the WRKY family (WRKY55, WRKY50, WRKY15, and WRKY2) was induced under drought stress [49]. In *Gossypium hirsutum*, WRKY25 (GhWRKY25) negatively regulates drought stress but positively regulates salt stress [50]. More experiments are required to explore the functions of WRKY TFs in plants.

C2H2-type zinc finger proteins belong to the major family of TFs that can be classified as osmotic stress-responsive genes [51]. C2H2 regulates plant responses to drought resistance through ABA-dependent and ABA-independent pathways [25]. Seven tomato C2H2 genes were shown by RNA sequencing to be specifically expressed under drought stress [52]. In this study, three C2H2 TFs were downregulated under drought stress, including StZAT11 (c70317.graph_c0). ZAT11 overexpression increases the elongation of primary roots in Arabidopsis, but the biological function of ZAT11 in drought tolerance remains unknown [53]. We found that *S. tonkinensis* C2H2 TFs may play negative roles in drought tolerance.

StAGL80 was the only MADS TF that was differentially regulated by drought treatment in *S. tonkinensis*. AGL80 is required for central cell and endosperm development in Arabidopsis [54, 55]. However, the functions of AGL80 in the drought stress response of plants remain to be determined.

### Quinolizidine alkaloid biosynthesis under drought stress

Secondary metabolites (such as flavonoids and alkaloids) accumulate in plants during drought stress [56–58]. Alkaloids, which are widely distributed throughout the plant kingdom, are derived from lysine and further subdivided into piperidine, quinolizidine, indolizidine, and lycopodium alkaloids [59]. Quinolizidine alkaloids occur mainly in the family Leguminosae, especially in the genera *Lupinus*, *Baptisia*, *Thermopsis*, *Genista*, *Cytisus*, and *Sophora* [59]. Matrine is a quinolizidine alkaloid and is the main medicinally active ingredient of *S. tonkinensis*. The molecular mechanisms underlying the biosynthesis of quinolizidine alkaloids are not well understood.

In *S. tonkinensis*, LDC and CAO catalyse the first two steps in the conversion of lysine into 5-aminopentanal. 5-Aminopentanal undergoes a series of reactions to produce matrine, which can be oxidized into oxymatrine. Through mRNA sequencing, we found that two unigenes, c87510.graph_c0 (encoding LDC) and c100937.graph_c1 (encoding CAO), were upregulated under drought treatment, indicating that they may participate in the drought response of *S. tonkinensis*. Drought stress significantly promoted the accumulation of matrine and oxymatrine, especially in MDT. However, the contents of matrine and oxymatrine in SDT were lower than those in MDT, probably because severe drought may cause irreversible damage to plants. The miRNAs targeting these two unigenes were not identified by miRNA sequencing. Hence, the role of miRNAs in regulating quinolizidine alkaloid biosynthesis needs to be further studied.

### miRNAs modulate gene expression under drought stress

In plants, miRNAs regulate gene expression mainly by cleaving targeted mRNAs or transcriptional inhibition [16, 17]. The regulation of gene expression mediated by miRNAs and their target mRNAs provides a sophisticated mechanism whereby plants respond to stress responses [16]. Through small RNA sequencing, 368 miRNAs and greater than ten thousand target genes of miRNAs were found in *S. tonkinensis*. Drought treatment resulted in the identification of 17 and 27 DEMs in MDT and SDT, respectively. The KEGG analysis of the target genes of those DEMs showed that the “plant hormone signal transduction” pathway was enriched in both MDT and SDT. These genes, including AUX1, CRE1, APF, and ETR, play important roles in the auxin, cytokine, ABA, and ethylene signal transduction pathways. Plant hormones play a key role in signalling networks involved in plant development and stress responses [60, 61]. The identified plant hormone-associated genes were targeted and regulated by different miRNAs in response to drought. For instance, the CRE1 gene (c104402.graph_c1), which is related to cytokine signalling, was targeted by a novel miRNA (unconservative_c98521.graph_c0_46983) in response to drought stress in our study. The ABF gene (c86591.graph_c0), which is related to ABA signalling, was targeted by 4 known miRNAs (Sto-miR159n, Sto-miR166u, Sto-miR396k, and Sto-miR396m). However, among these miRNAs, only Sto-miR159n was differentially regulated by drought treatment. The results indicated the significant involvement of plant hormone signal transduction genes and miRNAs in regulating the drought response of *S. tonkinensis*.

The miRNAs Sto-miR398b and Sto-miR5225 were co-downregulated in MDT and SDT. The downregulation of miR398 in *S. tonkinensis* was consistent with results reported in tobacco and wheat but was not consistent with results reported in *M. truncatula* and wild emmer wheat [62, 63]. miR5225 is induced by drought in drought-tolerant apple trees [64]. Sto-miR482d was co-upregulated in MDT and SDT, in contrast to previous results showing that miR482 is downregulated in alfalfa roots under drought stress [65]. These results suggest that most drought-responsive miRNAs play important, yet different, roles in the response to drought in different plants.

The DEM and differentially expressed mRNA datasets were analysed to construct the miRNA-mRNA regulatory network model under drought stress. A total of 11 and 26 differentially expressed miRNA-mRNA pairs were identified in MDT and SDT, respectively. Both positive and negative correlations were found in our results. The most common regulatory pattern was a positive correlation of upregulated miRNAs targeting upregulated mRNAs, as observed between upregulated miR156z and its 4 upregulated target genes (c100636.graph_c0, c103156.graph_c2, c94398.graph_c0, c97480.graph_c1). Positive correlations maintain a proper balance between miRNA and target mRNA expression levels when needed, suggesting the involvement of finely tuned mechanisms to modulate gene expression through a miRNA-mediated feedback loop [66]. In contrast, a negative correlation was found between downregulated Sto-miR156n and upregulated c103497.graph_c0, suggesting that miRNAs from the same miRNA families can display different expression patterns and play different roles in regulating the expression of drought-responsive mRNAs. The putative mRNA-miRNA interactions identified in this study may play important, higher-order molecular genetic regulator roles (i.e., posttranscriptional) in the drought stress responses of *S. tonkinensis*.

## Conclusions

In the present study, we generated mRNA and small RNA sequencing datasets from *S. tonkinensis* roots under MDT and SDT and performed a comprehensive analysis of drought-responsive genes and miRNAs. mRNA sequencing revealed hundreds of DEGs under drought stress. According to the KEGG analysis, the DEGs included genes related to phenylpropanoid biosynthesis, nitrogen metabolism, protein processing in the endoplasmic reticulum, linoleic acid metabolism, sugar metabolism, and quinolizidine alkaloid biosynthesis. Our findings suggested that the differential regulation and expression of TFs plays a central role in drought stress responses. In addition, our analysis also identified miRNAs that may play important roles in the response to drought stress in *S. tonkinensis* roots. Many of the miRNAs and their target genes were involved in the regulation of plant hormone signal transduction, spliceosomes, and ribosomes. The integrated analysis of miRNA-mRNA interaction networks also revealed many finely tuned mechanisms of drought stress tolerance. Taken together, our results suggest that *S. tonkinensis* implements diverse mechanisms to modulate its responses to drought stress.

## Materials and methods

### Plant materials

Mature seeds were collected from one cultivated *S. tonkinensis* plant grown in Huanjiang district, Guangxi, China, with permission granted by the owner. The plant materials were formally identified by Professor Kunhua Wei and have been deposited in the Germplasm Repository of the Guangxi Botanical Garden of Medicinal Plants, Nanning, China, with voucher number YYZW20190015. The seeds were sown in pots containing a mixture of soil and vermiculite (2:1, w/w) and incubated at 26 °C/20 °C under a 14 h light/10 h dark photoperiod, in a laboratory of the Guangxi Botanical Garden of Medicinal Plants. After germination, 30-day-old seedlings of *S. tonkinensis* were used in this study. The seedlings with the same development status were divided into three groups receiving different irrigation treatments, with 60 seedlings in each treatment. The plant heights of the selected seedlings were approximately 8 cm, and the stem diameters were approximately 2 mm. In the control group, a 75-80% available soil water (ASW) content was maintained for 10 days. In the mild drought treatment and the severe drought treatment, 55-60% and 30-35% ASW contents, respectively, were maintained for 10 days. The maintenance of ASW contents was conducted by measuring water potential. At the end of the experiment, plant roots were harvested, immediately frozen in liquid nitrogen, and stored at − 80 °C for subsequent sequencing analyses. The fresh weight and dry weight of the roots were determined using an electronic scale. Plants were rinsed with distilled water to clean them. After drying, the plants were weighed to determine the fresh weight of the roots. The dry weight of the roots was obtained by incubating samples at 65 °C for 5 days.

### Determination of soluble sugar, soluble protein, and malondialdehyde contents and antioxidant enzyme activities

The physiological indices of the contents of soluble sugar, soluble protein, and malondialdehyde (MDA), were determined with enzyme-linked immunosorbent assay (ELISA) kits (cat. ml670187, ml680403, and ml505357, respectively; Shanghai Enzyme-linked Biotechnology, Shanghai, China). The activities of three antioxidant enzymes, peroxidase, superoxide dismutase, and catalase, were also determined with ELISA kits (cat. ml607323, ml606325, and ml202784, respectively; Shanghai Enzyme-linked Biotechnology, Shanghai, China). Briefly, root samples from different irrigation treatments were frozen in liquid nitrogen immediately after harvesting and stored at − 80 °C. Approximately 50 mg of roots was added to 450 μl of phosphate-buffered saline (PBS) (pH 7.2-7.4), and homogenization with a grinder was conducted in an ice bath. The homogenate was centrifuged at 15,000 g for 20 min at 4 °C, and the supernatant was collected and used in the subsequent ELISAs according to the product instruction manuals. The experiment was repeated three times.

### Determination of genistein and maackiain contents

A portion of 0.05 g dried roots was added with 1 ml methanol and then ground. The solution was ultrasonicated for 50 min and centrifuged at 12000 rpm for 10 min to obtain the supernatant. The supernatant was then dried and reconstituted with 0.5 ml methanol. The solution was filtered through a 0.22 μm filter prior to HPLC. The HPLC analysis was carried out on a Wufeng LC-100 series HPLC system (Wufeng, Shanghai, China) with a C18 reversed-phase chromatography column (5 μm, 4.6 × 250 mm). The temperature was maintained at 30 °C by a CTO-20 AC column controller (Shimadzu, Kyoto, Japan). An aqueous methanol stock solution containing genistein and maackiain reference substance was prepared and was diluted to the respective appropriate concentrations to produce calibration curves. Each sample was performed in three biological replicates and three technical replicates.

### Determination of matrine and oxymatrine contents

The contents of matrine and oxymatrine were determined by HPLC according to the guidelines of the Chinese Pharmacopoeia (edition 2015). Briefly, 0.5 g of dried roots was ground and placed in a 100 ml centrifuge tube containing 50 ml chloroform/methanol/ammonia solution (40:10:1). The mixture of sample powder and solution was ultrasonically crushed for 30 min, and then filtered. A total of 25 ml of the filtrate was accurately measured. The filtered extract was then dried and transferred to a 25 ml volumetric flask using methanol and was filtered through a 0.45 μm filter prior to HPLC. The HPLC analysis was carried out on a Waters HPLC alliance e2695 separating module (Waters, Milford, MA, USA) with the column Agilent Polaris NH2 (5 μm, 4.6 × 250 mm). An aqueous methanol stock solution containing matrine and oxymatrine reference substance was prepared and was diluted to the respective appropriate concentrations to produce calibration curves. Each sample was performed in three biological replicates and three technical replicates.

### Transcriptome sequencing and quality control

For each treatment, three independent biological replicates were used to extract total RNA with an RNAprep Pure Plant kit (Tiangen, Beijing, China). The total RNA of each sample was quantified and qualified via Agilent 2100 Bioanalyzer (Agilent Technologies, Palo Alto, CA, USA), NanoDrop (Thermo Fisher, Carlsbad, CA, USA), and 1% agarose gel analyses. Thereafter, a total of 3 μg of RNA per sample was used as the input material for RNA sample preparation. Sequencing libraries were generated using the NEBNext®Ultra™ RNA Library Prep Kit for Illumina® (NEB, Ipswich, MA, USA) following the manufacturer’s protocol, and index codes were added to attribute the sequences to each sample. The libraries were then sequenced on the Illumina HiSeq X ten platform (Illumina, San Diego, CA, USA), and paired-end reads were generated. Next, clean data (clean reads) were obtained by removing reads containing adapters, reads containing poly-N sequences and low-quality reads from the raw data. In addition, the Q20, Q30, GC content, and sequence duplication level were calculated from the clean data. All downstream analyses were based on high-quality clean data.

### Transcriptome data analysis

The clean reads were de novo assembled using Trinity v2.5.1 software [67]. Gene functions were annotated based on the following databases: National Center for Biotechnology Information (NCBI) non-redundant protein sequences (NR); Protein family (Pfam); euKaryotic Orthologous Groups (KOG); Clusters of Orthologous Groups (COG); evolutionary genealogy of genes: Non-supervised Orthologous Groups (eggNOG); Swiss-Prot (a manually annotated and reviewed protein sequence database); Kyoto Encyclopedia of Genes and Genomes (KEGG); and Gene Ontology (GO). Differential expression analysis between the different irrigation treatments was performed using the DESeq R package (version 1.10.1) [68]. The obtained *p*-values were adjusted using the Benjamini and Hochberg method to control the false discovery rate (FDR). An FDR ≤ 0.01 and a log_2_ fold-change (|log_2_ FC|) ≥ 1 were set as the thresholds for significantly differential expression. For TF prediction, the set of Arabidopsis TFs in Plant TFDB 3.0 [69] was used as the reference TF database. The TF prediction algorithm HMMER 3.0 [70] was used to identify TFs and assign genes to different families.

### Small RNA sequencing and quality control

For small RNA sequencing, 5 μg of total RNA was used to construct a sRNA library according to the manual of the NEBNext® Multiplex Small RNA Library Prep Set for Illumina (NEB, MA, USA). Library quality was assessed on an Agilent Bioanalyzer 2100 system with DNA High Sensitivity Chips. The libraries were then sequenced on the Illumina HiSeq X Ten platform (Illumina, San Diego, CA, USA). After obtaining the raw reads, quality control checks were performed using a custom Perl script to obtain clean reads, which involved the removal of reads containing adapters, reads containing poly-N sequences and low-quality reads from the raw data. Thereafter, the reads were trimmed and cleaned by removing sequences smaller than 18 nt or longer than 30 nt.

### Small RNA sequencing data analysis

Using Bowtie software (v1.0.0) [71], the clean reads were compared with the Silva database, GtRNAdb database, Rfam database, and Repbase database to remove noncoding RNAs (rRNA, tRNA, snRNA, snoRNA). The remaining reads were used to detect known miRNAs from miRbase (http://www.mirbase.org/) and predict novel miRNAs by comparison with the unigenes obtained from the transcriptome assembly, using miRDeep2 software (v2.0.5). The expression level of each identified miRNA was estimated according to the transcripts per million (TPM) value by applying following normalization formula: normalized expression = mapped readcount/total reads*1,000,000. Differential expression analysis between two groups was performed using the DESeq R package (version 1.18.0) with thresholds of a corrected *p*-value < 0.05 and an absolute fold-change value > 1.5 [68]. The prediction of miRNA target genes was performed using Target Finder software (v1.6).

### Gene ontology annotation, KEGG pathway analysis, and miRNA-mRNA network analysis

The GO term annotation of the differentially expressed genes (DEGs) and miRNA target genes was conducted according to biological processes (BPs), cellular components (CCs), and molecular functions (MFs). KOBAS software was used to test the statistical enrichment of the DEGs and miRNA target genes in KEGG pathways [72]. A network showing the miRNA-mRNA relationships between differentially expressed miRNAs (DEMs) and their target genes was constructed using Cytoscape, as previously described [23, 73].

### qPCR validation of transcriptome and small RNA sequencing results

The expression of selected genes and miRNAs was determined by qRT-PCR. Approximately 0.5 μg of total RNA was reverse transcribed using a TUREscript 1st Strand cDNA Synthesis Kit (Aidlab, Beijing, China). Gene-specific mRNA primers for were designed for 11 randomly selected genes based on the obtained sequences. The actin gene (c103018.graph_c0) was used as the reference gene. qRT-PCR was performed using 2 × SYBR Green Supermix (Bio-Rad, CA, USA) on a qTower 2.2 instrument (AnalytikJena, Jena, Germany) according to the manufacturer’s protocol. Specific miRNA primers were designed based on the sequences of 8 selected mature miRNAs. The 18S gene was chosen as an endogenous internal control. The primers used for qRT-PCR are listed in Table S1. The qRT-PCR assay was performed with three biological replicates, and relative expression levels were calculated based on the 2-ΔΔCt method [74].

### Statistical analysis

Multiple comparison testing was performed using one-way ANOVA followed by the least significant difference (LSD) test. Statistical analyses were performed using SPSS software version 22.0. The results are expressed as the means ± standard errors of the means. *p* < 0.05 was considered statistically significant. Student’s t-test was used to calculate the *p*-value of the significance of root weight and qRT-PCR assay results.

## Supplementary Information

**Additional file 1: Figure S1.** GO enrichment analysis of co-upregulated (A) and co-downregulated (B) unigenes.

**Additional file 2: Figure S2.** Genistein and maackiain contents in CK, MDT and SDT.

**Additional file 3: Figure S3.** Matrine and oxymatrine contents in CK, MDT and SDT.

**Additional file 4: Table S1.** Primers used for qRT-PCR.

**Additional file 5: Table S2.** Differentially regulated genes in CK vs. MDT and CK vs. SDT.

**Additional file 6: Table S3.** Known and novel miRNAs identified in the study.

## Data Availability

The datasets generated and analysed during the present study are available from the corresponding author on reasonable request. All the sequence data generated in this research were deposited in the Sequence Read Archive database (www.ncbi.nlm.nih.gov/sra) at NCBI under accession number: PRJNA680893. (https://www.ncbi.nlm.nih.gov/sra/?term=PRJNA680893).

## Abbreviations

*S. tonkinensis*: Sophora tonkinensis
DEGs: Differentially expressed unigenes
DEMs: Differentially expressed miRNAs
FC: Fold-change
TPM: Transcripts per million
FPKM: Fragments per kilobase of exon per million fragments mapped
FDR: False discovery rate
LSD: Least significant difference
TF: Transcription factor
CK: Control treatment
MDT: Mild drought treatment
SDT: Severe drought treatment
GO: Gene Ontology
COG: Clusters of Orthologous Groups
NR: non-redundant protein sequences
NCBI: National Center for Biotechnology Information
Pfam: Protein family
KOG: uKaryotic Orthologous Groups
eggNOG: Non-supervised Orthologous Groups
KEGG: Kyoto Encyclopedia of Genes and Genomes
CHS: Chalcone synthase
LDC: Lysine decarboxlylase
CAO: Copper amine oxidase
MDA: Malondialdehyde
qRT-PCR: Quantitative reverse-transcription PCR
COMT: Caffeic acid 3-O-methyltransferase
ABA: Abscisic acid
ASW: Available soil water
ELISA: Enzyme-linked immunosorbent assay
HPLC: High-performance liquid chromatography
BPs: Biological processes
CCs: Cellular components
MFs: Molecular functions
