# Supplementary figures and images for: Integrated transcriptome and small RNA sequencing analyses reveal a drought stress response network in Sophora tonkinensis
Source: BMC Plant Biol. 2021 Dec 2;21:566. doi: 10.1186/s12870-021-03334-6 (PMC8641164; doi:10.1186/s12870-021-03334-6)

A

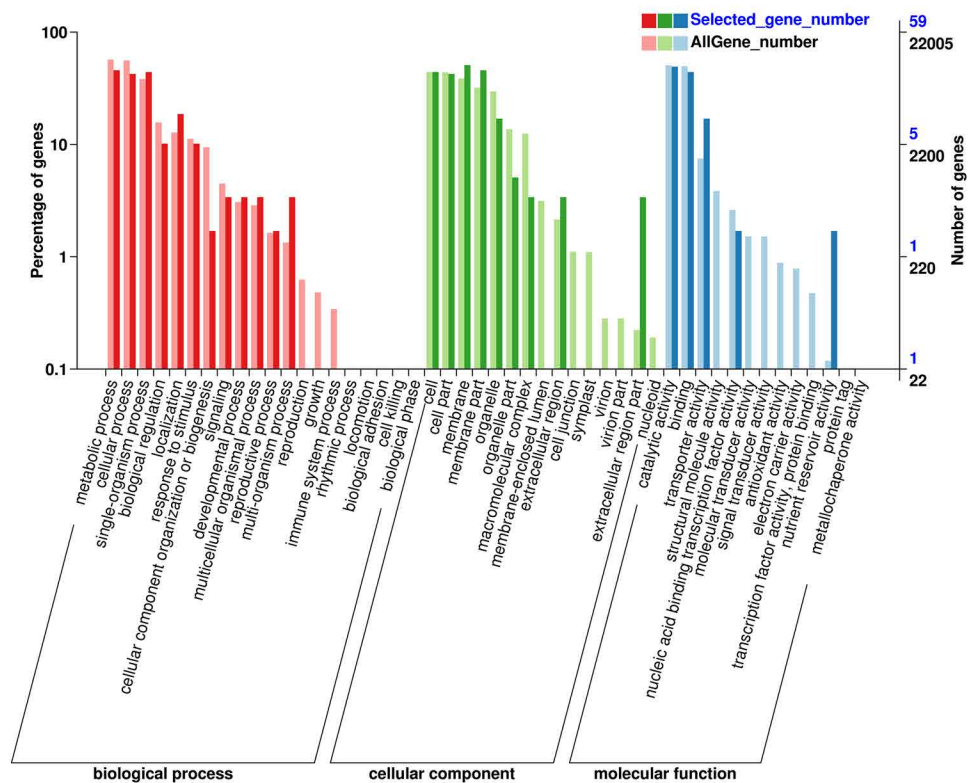

B

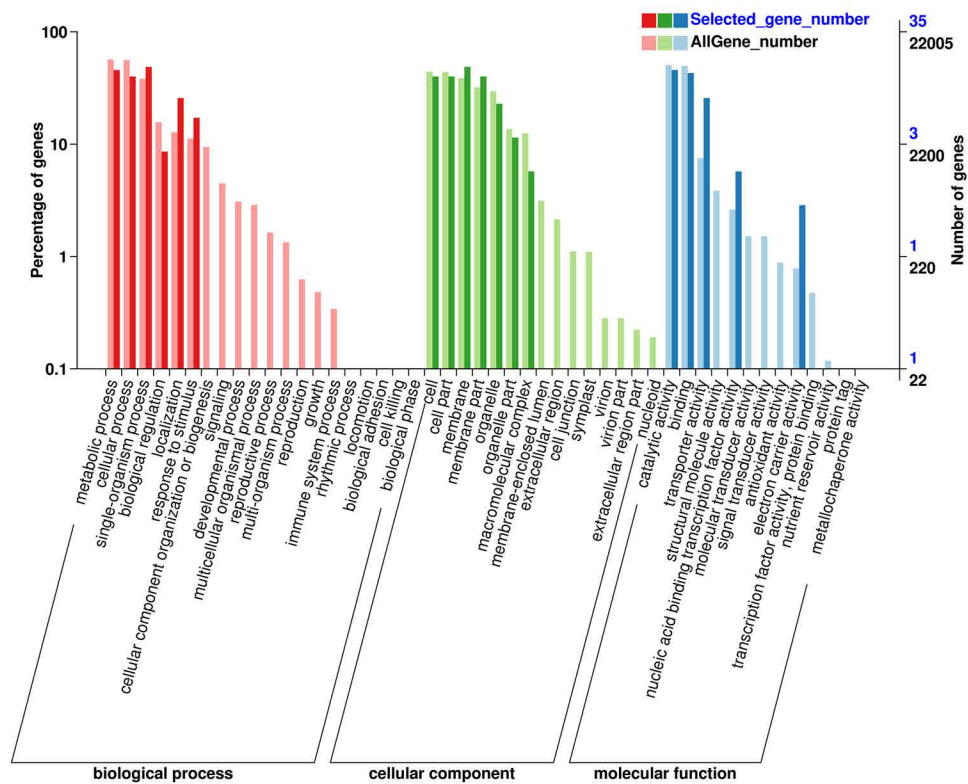

Supplement: Supplementary file 1 — Additional file 1: Figure S1. GO enrichment analysis of co-upregulated (A) and co-downregulated (B) unigenes. [file 12870_2021_3334_MOESM1_ESM.pdf]

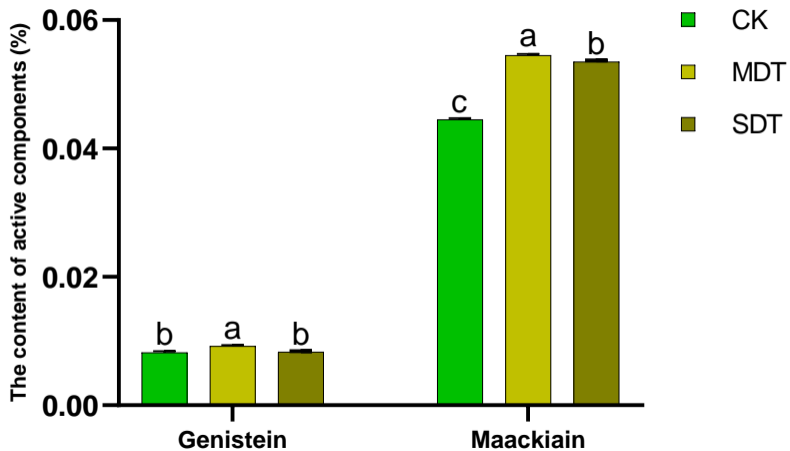

Supplement: Supplementary file 2 — Additional file 2: Figure S2. Genistein and maackiain contents in CK, MDT and SDT. [file 12870_2021_3334_MOESM2_ESM.pdf]

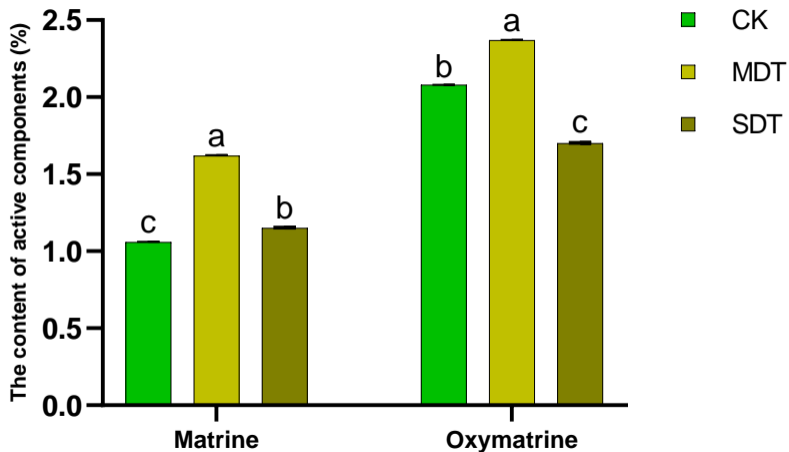

Supplement: Supplementary file 3 — Additional file 3: Figure S3. Matrine and oxymatrine contents in CK, MDT and SDT. [file 12870_2021_3334_MOESM3_ESM.pdf]
